# Supplementary material for: Patterns of Intron Gain and Loss in Fungi
Source: PLoS Biol. 2004 Nov 30;2(12):e422. doi: 10.1371/journal.pbio.0020422 (PMC532390; doi:10.1371/journal.pbio.0020422)
Supplement: Table S1 — Also available at http://genes.mit.edu/NielsenEtAl/. (4.3 MB ZIP). [file pbio.0020422.st001.zip › NielsenEtAl/html/439.html]

AN4714.1.NCU00605.1.MG02856.1.FG09413.1


```
 CLUSTAL W (1.82) Multiple Sequence Alignments - Introns Inserted


Sequence 1: NCU00605.1	510 aa
Sequence 2: FG09413.1	523 aa
Sequence 3: MG02856.1	524 aa
Sequence 4: AN4714.1	515 aa
Alignment Length: 542 aa
Number Identitical Residues: 218 aa
Alignment Score (without introns) 11050


MG02856.1 	MASSFITRATSDSRFQFAATALLSGAVAAGAILSYQRVAKEERLSKLKSSIPAPDEDGDM
NCU00605.1	MSSFLS-NSSNQSKLQLATVALASAAVTAGAIYGYQQSRHGERLNRLKKSIPNPAGDAEP
FG09413.1 	MSSFLSSETLSNPRFQLFAAAAFSGATVASLILGYQALEREERVYELKNSIPADDPNLHP
AN4714.1  	MASWIQ-RQNNSHQVQLAATAVLSGAAVAGAILGFQKYRRREAVKRLKASIPTIDEKHRA
          	*:* :  .  .. :.*: :.*  *.*..*. * .:*   : * : .** ***    .   

MG02856.1 	SRS-----QQ0LTDFG-------ATLPTNPDKEDLRNAALARRAMAGDYDEE~LILEQLA
NCU00605.1	EL------QR0VTRQ--------GPVPK-LDKEDEHNQALAHRAQNGDFDDE~LILEQLA
FG09413.1 	VLSSCFFSKN~TTDQSQLNNFGGSSAPP-VDKEDARNQAMARRAQAGDFDEE~LILEQLA
AN4714.1  	ES--------~LNEFG------AAVPGPYWSKEDERGAALARRAQEGDYDEE1LILEQLA
          	            .  .      ..      .*** :. *:*:**  **:*:* *******

MG02856.1 	RNAVFLGDEGMTKLRRSFVIVVGCGGVGSHCATSLARSGVGRIRLIDFDNVSLSSLNRHA
NCU00605.1	RNRVFLGDEGLAKLRNSFVVIVGCGGVGSHAATTLARSGVSKLRLIDFDQVTLSSLNRHA
FG09413.1 	RNRVFLTDEGLDKLRNSFVIVVGCGGVGSHCTAALARSGVSKIRLIDFDQVTLSSLNRHA
AN4714.1  	RNRVFLKDEGLAKLRDAFIIVVGCGGVGSHAVASLARSGVSKIRLIDFDQVTLSSLNRHA
          	** *** ***: *** :*:::*********..::******.::******:*:********

MG02856.1 	VATLADVGTQKTACLKRRLVAIAPWIRFDLCTEKFDSEAEERLLGPWAED----GAKPDF
NCU00605.1	VATLADVGIPKVQCLQRRLIAITPWVRFDLRLQKFDGSVAPELLGAWEKD----GQMPDF
FG09413.1 	VATLADVGIPKVQCLERRMMAIAPWVKFDLRQEQFNENVAERLLRPWGDD----GRAPDF
AN4714.1  	LATLADVGTPKVHCIRRRLQQIVPWVKFDCRNELFGASAADDLLAPWTLDDADKGQKPVY
          	:*******  *. *:.**:  *.**::**   : *. ..   ** .*  *.:..*  * :

MG02856.1 	VVDAIDNIETKVELLRYCHEHKIPVISSMGAACKGDPTRVMVGDVGVSTDDPLSRATRRR
NCU00605.1	VIDAIDNIDSKVELLKYCYDNNLPVISSMGAGTKSDPTRIMVGDIGTSTDDGLSRATRRR
FG09413.1 	VIDAIDNIETKVSLLEYCHKNNLPVISAMGAGCKSDPTRIIVGDIGSSKDDGLSRATRRK
AN4714.1  	VLDCIDNIQSKVELLHYCHSHSIPVISSMGAGCKSDPTRVMITDMSVSSDDRLSRSTRRR
          	*:*.****::**.**.**:.:.:****:***. *.****::: *:. *.** ***:***:

MG02856.1 	LKLLGITSGIPVVFSTEQRGEGKAELLPLSEEEFQKGSVGDLGVLPDFRVRILPVLGTMP
NCU00605.1	LKLLGVTSGIPVVYSTEKMGEGKAALLPLPEEEFKKGDVGDLAALPDFRVRILPVLGTMP
FG09413.1 	LKLKGITSGVPVVYSTETAGAGKAELLPLPEEEFQKGSVGDLAAMPNFRVRILPVLGTMP
AN4714.1  	LKLLGVTTGIPVVFSTEKPGPGKATLLPLAEEEFAKGEVGELSVLPDFRSRILPVLGTMP
          	*** *:*:*:***:***  * *** ****.**** **.**:*..:*:** **********

MG02856.1 	AVFGYTAANHVILSITG1YPTDYAPG-KSRDKLYDQALASLQGTEAKVVRSFNGGNADIV
NCU00605.1	AVFGYTVANHVILKISG1YPLDYIPQ-KGRDKMYDGIQAFVQASEEKIIRAVTGGPRELC
FG09413.1 	AIFGLTVANHVILSITG~YPLDYVPA-KGREKMYEGMLATIQSSEEKLARMTYEGDT---
AN4714.1  	AVFGYTLANHVICEISE~YPTDYSMGGKGKDKLYDTVHAQLLVTLERLARAESESGTQP-
          	*:** * ***** .*:  ** **   .*.::*:*:   * :  :  :: *    .  .  

MG02856.1 	VGLRTPLSPGDIAFMIEEVFRGRSAITGIATRLVLVRWRRPATDPLMVIEGAPDDDGPQK
NCU00605.1	IGLKVPIQPCEVSFLVEDIYKAKSAVTGLPTKLVLIRWQKPTRDILIRIGEGADE-----
FG09413.1 	VGLKVPITTGDVAFLSEELYRGRSAISGIPTKLVLIRWEKPEGPSMTTLGEGKDT-----
AN4714.1  	IGLRLPMSRDDVIYLVDEIWRGKSVVTGLPSRLALTLWNRPSNGFKPDPQWEKEG-----
          	:**: *:   :: :: :::::.:*.::*:.::*.*  *.:*            :      

MG02856.1 	SAPAPQKSTRLRLSDLVCMTKDEAMVHLREVLLAGKQPEEMYDAETVERVNMRLSEAAGY
NCU00605.1	-----QKSSDLKLSELVCMTKDEATRHQREVLLGEKTLEELYDAEIIEKVAKRQEEIKLY
FG09413.1 	-----QKCSTVKLRDLVLMTKEEATRHEKGIFKEGKSLDEVYDAETLARVEEKRATAEKY
AN4714.1  	-----QILIPFKPEDLVLMTKEEATRHEKEVLMGGKKVEDLYSEEIIQKVNQRQKEMAYY
          	     *    .:  :** ***:**  * : ::   *  :::*. * : :*  :      *

MG02856.1 	EKFR-
NCU00605.1	EKYR-
FG09413.1 	EAFRS
AN4714.1  	EQFR-
          	* :*
```
